# Development of Pentaplex Reverse Transcription Droplet Digital PCR Assay for Simultaneous Detection and Absolute Quantification of HIV‐1, HIV‐2, HCV, and HBV With Internal Control

**DOI:** 10.1002/elps.70098

**Published:** 2026-05-27

**Authors:** Soo Yeon Lim, Un Na Koh, Ah Leum Kim, Yebin Kim, Ga Eun Kim, Si‐Keun Lim

**Affiliations:** ^1^ Department of Forensic Sciences Sungkyunkwan University Suwon Republic of Korea; ^2^ ID‐Cell Forensics Co Sungkyunkwan University Suwon Republic of Korea; ^3^ Convergence Bio Forensic Institute (CBFI) Biomedical Institute for Convergence at Sungkyunkwan University (BICS), Sungkyunkwan University Suwon Republic of Korea

**Keywords:** droplet digital PCR, hepatitis B virus (HBV), hepatitis C virus (HCV), human immunodeficiency virus (HIV), pentaplex RT‐ddPCR, QX600, viral load quantification

## Abstract

This study presents the development of a pentaplex reverse transcription droplet digital polymerase chain reaction (RT‐ddPCR) assay for the simultaneous detection and absolute quantification of human immunodeficiency virus (HIV)‐1, HIV‐2, hepatitis C virus (HCV), hepatitis B virus (HBV), and an internal control. These viruses are important blood‐borne pathogens for diagnostics, blood screening, and the validation of reference materials. Commercial kits used for blood screening allow multiplex detection but rely on relative, cycle threshold‐based quantification approaches; consequently, the results may vary depending on reference materials and analytical conditions, which limit interlaboratory comparability. In addition, conventional antigen–antibody assays and single‐target quantitative polymerase chain reaction (qPCR) methods have limited multiplexing capability and require standard curves, restricting their broader application. The assay demonstrated 95% limit of detection (LoD) values ranging from 2.42 to 6.65 copies/reaction across targets with high specificity. Validation following the principles outlined in International Organization for Standardization (ISO) 20395:2019 demonstrated the linearity, sensitivity, and precision of the assay, with low coefficients of variation indicating high reproducibility. When evaluated using a nanowell plate‐based system, the assay showed comparable performance without statistically significant differences, thereby demonstrating platform scalability. The applicability of the assay was further confirmed using externally sourced pathogen resources, including human plasma–derived HIV‐1–positive materials under conditions resembling clinical specimens, demonstrating reliable detection beyond international reference materials. This study introduces a robust multiplex platform for diagnostics, blood screening, and quality control of reference materials. The assay integrates qualitative and quantitative analysis within a single workflow, enabling efficient multi‐target detection.

## Graphical abstract

Development of Pentaplex reverse transcription droplet digital PCR assay for simultaneous detection and absolute quantification of HIV‐1, HIV‐2, HCV, and HBV with internal control. The channel‐resolved multiplex strategy enables high sensitivity, robust quantification, and scalable digital PCR workflows across platforms.

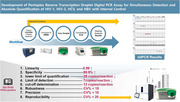

**Abbreviations**
BLAST: basic local alignment search tool
CI: confidence interval
CV: coefficient of variation
ddPCR: droplet digital polymerase chain reaction
dPCR: digital polymerase chain reaction
HBV: hepatitis B virus
HCV: hepatitis C virus
HIV: human immunodeficiency virus
HIV‐1: human immunodeficiency virus type 1
HIV‐2: human immunodeficiency virus type 2
IC: internal control
ISO: International Organization for Standardization
IVD: in vitro diagnostics
LLoQ: lower limit of quantification
LoD: limit of detection
NCBI: National Center for Biotechnology Information
NIBSC: National Institute for Biological Standards and Control
pgRNA: pregenomic RNA
qPCR: quantitative polymerase chain reaction
RT‐ddPCR: reverse transcription droplet digital polymerase chain reaction
RT‐qPCR: reverse transcription quantitative polymerase chain reaction
RUO: research‐use‐only
SD: standard deviation
WHO: World Health Organization

## 1 Introduction

Human immunodeficiency virus (HIV), hepatitis B virus (HBV), and hepatitis C virus (HCV) are major blood‐borne pathogens associated with high global prevalence and mortality [1, 2, 3]. These viruses frequently cause chronic infections, prompting the World Health Organization (WHO) to establish a global strategy aimed at their elimination by 2030 through expanded prevention, testing, and treatment efforts [4].

Due to their significant disease burden and transfusion‐associated risk, HIV, HBV, and HCV are key analytical targets in diagnostics, blood screening, and the development of biological reference materials [5, 6, 7, 8, 9]. Since the introduction of nucleic acid amplification‐based screening tests in the late 1990s, the risk of transfusion‐transmitted infections has been substantially reduced [10, 11]. Currently, most blood centers rely on commercial multiplex nucleic acid tests that enable simultaneous qualitative detection of these viruses [12, 13, 14, 15]. However, such assays are primarily designed for screening purposes and do not support absolute quantification, limiting their utility for virological research, assay standardization, and reference material evaluation.

Quantitative viral load measurement remains essential for clinical diagnostics and laboratory applications and is typically performed using singleplex quantitative polymerase chain reaction (qPCR) assays based on standard curves. Although qPCR has long been the mainstream method for viral detection, its reliance on standard curves and susceptibility to PCR inhibitors limit quantitative comparability and robustness, and multiplex systems capable of simultaneously quantifying multiple blood‐borne viruses within a single workflow remain limited [16].

Digital PCR (dPCR) overcomes key limitations of qPCR by enabling absolute quantification without the need for standard curves while providing high sensitivity and robustness against PCR inhibitors [17]. The droplet digital PCR (ddPCR) platform selected for the development of this assay replaces traditional wells with sub‐nanoliter‐sized droplets. Approximately 20 000 partitions were generated using microfluidic technology [18]. Early ddPCR‐based multiplex assays primarily relied on two‐color fluorescence systems and probe or primer concentration modulation to expand multiplexing capacity, approaches that are technically complex and prone to reduced specificity in inhibitor‐rich samples. Recent advances in ddPCR instrumentation, including six‐ or seven‐color fluorescence detection, now enable high‐order multiplex analysis within a single reaction; however, such capabilities remain underutilized in viral diagnostics and require further methodological development [16, 19].

**FIGURE 1:**
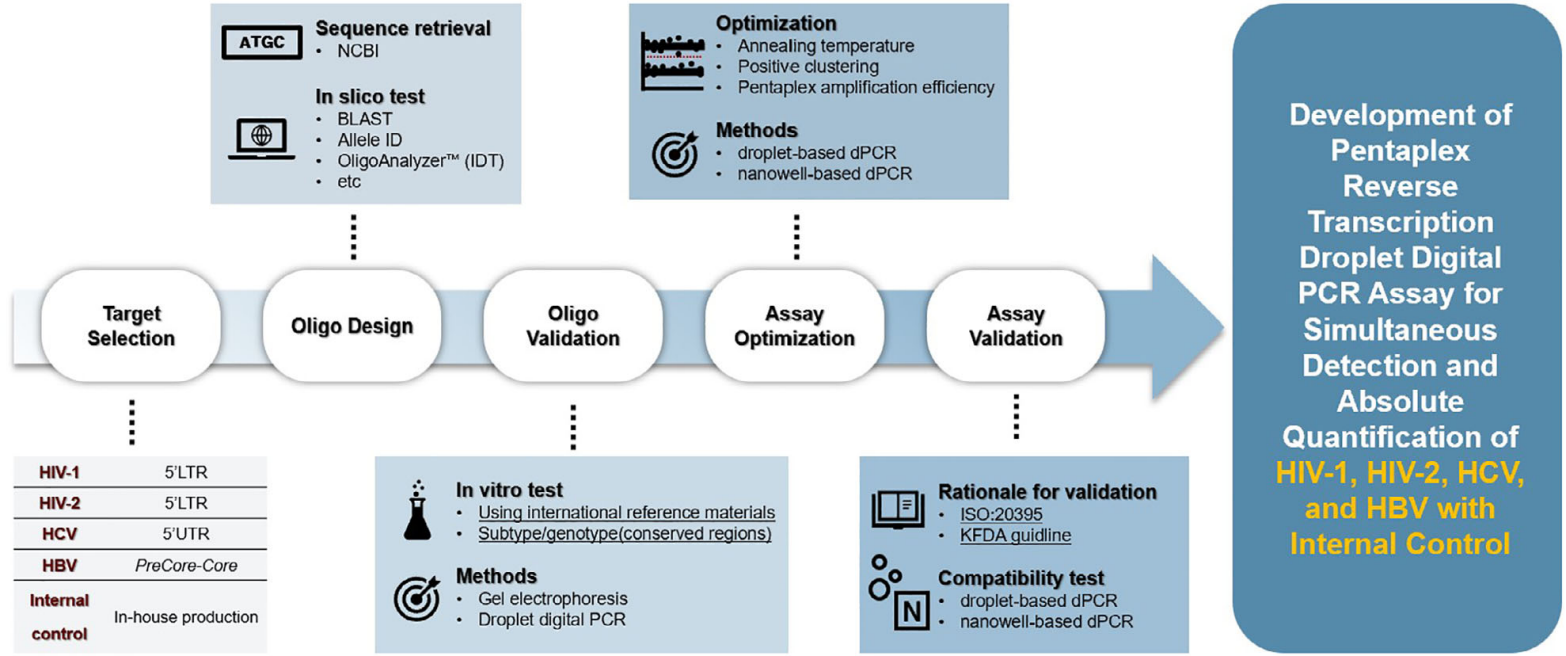
Workflow for the development and validation of a unified pentaplex reverse transcription droplet digital PCR assay for simultaneous detection and absolute quantification of HIV‐1, HIV‐2, HCV, and HBV. BLAST, basic local alignment search tool; dPCR, digital polymerase chain reaction; HBV, hepatitis B virus; HCV, hepatitis C virus; HIV, human immunodeficiency virus; NCBI, National Center for Biotechnology Information.

In this study, we developed a qualitative and quantitative integrated pentaplex RT‐ddPCR (reverse transcription droplet digital PCR) assay capable of simultaneously detecting HIV‐1, HIV‐2, HCV, HBV, and an internal control (IC). Assay performance was validated in terms of linearity, sensitivity, and precision in accordance with International Organization for Standardization (ISO) standards, and the analytical sensitivity of the proposed assay was further evaluated through comparison with a commercially available reverse transcription quantitative polymerase chain reaction (RT‐qPCR) kit targeting the same viral pathogens. Cross‐platform compatibility was also confirmed using both droplet‐based and the nanowell‐based Digital LightCycler dPCR System (Roche Diagnostics, Rotkreuz, Switzerland). In addition to international reference materials, the applicability of the assay was further assessed using externally sourced HIV‐1–derived materials, including human plasma–derived samples resembling clinical specimens. In addition, the assay was designed to allow flexible targeting of HBV nucleic acid species, enabling either RNA‐ or DNA‐focused analysis depending on the analytical objective [20, 21, 22]. By integrating multiplex qualitative detection with absolute quantification, this study aimed to provide a practical analytical framework for blood‐borne viral testing and reference material evaluation within a single workflow (Figure 1).

## 2 Materials and Methods

### 2.1 Sample Preparation

All viral nucleic acid samples used in this study consisted of international reference materials and pathogen resources obtained from external sources. International reference materials for HIV‐1, HIV‐2, HCV, and HBV were obtained from the National Institute for Biological Standards and Control (NIBSC, Blanche Lane, UK), with reference codes 16/194 (HIV‐1), 16/296 (HIV‐2), 18/184 (HCV), and 22/120 (HBV), respectively.

To evaluate assay performance across diverse subtypes and genotypes, additional reference panels were included: an HIV‐1 subtype panel from NIBSC (code: 12/224); HCV genotype 1a (0310‐0043), 1b (0310‐0046), 2a (0310‐0052), and 2b (0310‐0055) materials from SeraCare (Massachusetts, USA); and an HBV genotype panel from the Paul‐Ehrlich‐Institut (Langen, Germany; code: 5086/08).

In addition, 12 externally sourced pathogen resources were provided by the Global Infectious Disease Specimen Bank (GISB) at Institut Pasteur Korea, funded by the Ministry of Science and ICT, Republic of Korea (No. RS‐2024‐0040071). Originally collected in Côte d'Ivoire, these materials were derived from plasma of HIV‐1–infected individuals and were confirmed to be HIV‐1–positive by serological testing and HIV RNA quantitative RT‐PCR prior to distribution, thereby closely resembling clinical specimens in terms of biological matrix and potential PCR inhibitors, and were used to further assess assay applicability beyond international reference materials.

The IC RNA consisted of a 400 bp artificial, nonhomologous sequence synthesized in‐house (Table S1 in Supporting Information Data 1). Plasmid construction and in vitro transcription were performed by Bionics (Seoul, Republic of Korea). Lyophilized reference materials were reconstituted according to the accompanying worksheets, aliquoted into 1.5 mL microtubes for single use, and stored at −80°C until use.

### 2.2 RNA Preparation

All RNA used in the experiments was extracted using the QIAamp Viral RNA Mini Kit (QIAGEN, Hilden, Germany). Samples were prepared using two approaches: individual extraction for specificity evaluation and co‐extraction for pentaplex analysis. For individual extraction, each viral reference material was extracted separately together with IC. For combined extraction, a mixture of all the targets was extracted from a single tube.

**FIGURE 2:**
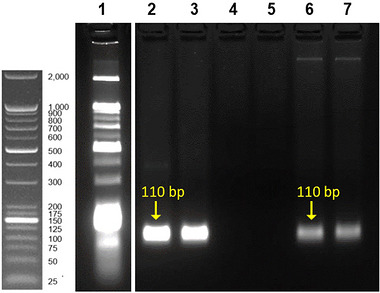
Effectiveness results of RNA clean‐up method with gel electrophoresis. Line 1: 25/100 bp Mixed DNA ladder, Line 2–3: HBV DNA + RNA (without DNase treatment), Line 4–5: HBV DNA + RNA (DNase treatment), Line 6–7: After cDNA conversion (HBV cDNA).

DNase treatment was applied to reduce potential DNA‐derived signals during HBV RNA analysis. RNA samples eluted in 60 µL were adjusted to a final volume of 100 µL with distilled water, followed by the addition of 2.5 µL of DNase I (Qiagen) and 10 µL of Buffer RDD (Qiagen). After incubation for 10 min at 20–25°C on a benchtop, RNA was purified using the RNeasy Mini Kit (Qiagen) according to the manufacturer's instructions. The effectiveness of this DNase‐based RNA clean‐up strategy was validated by gel electrophoresis (Figure 2), demonstrating the removal of HBV DNA signals following DNase treatment (Lanes 4–5) compared to untreated controls containing both HBV DNA and RNA (Lanes 2–3) and confirming successful downstream complementary DNA (cDNA) synthesis (Lanes 6–7), thereby supporting the specificity of pregenomic RNA (pgRNA) detection under high DNA background conditions.

### 2.3 cDNA Conversion

All samples used in the experiments were reverse‐transcribed into cDNA using RNA obtained from the previous steps. Reverse transcription was performed using the High‐Capacity cDNA Reverse Transcription Kit (Applied Biosystems, Foster City, CA, USA) according to the manufacturer's instructions. The resulting cDNA was either used immediately or aliquoted and stored at −20°C for up to 1 week when not used immediately.

### 2.4 Primers and Probes

**FIGURE 3:**
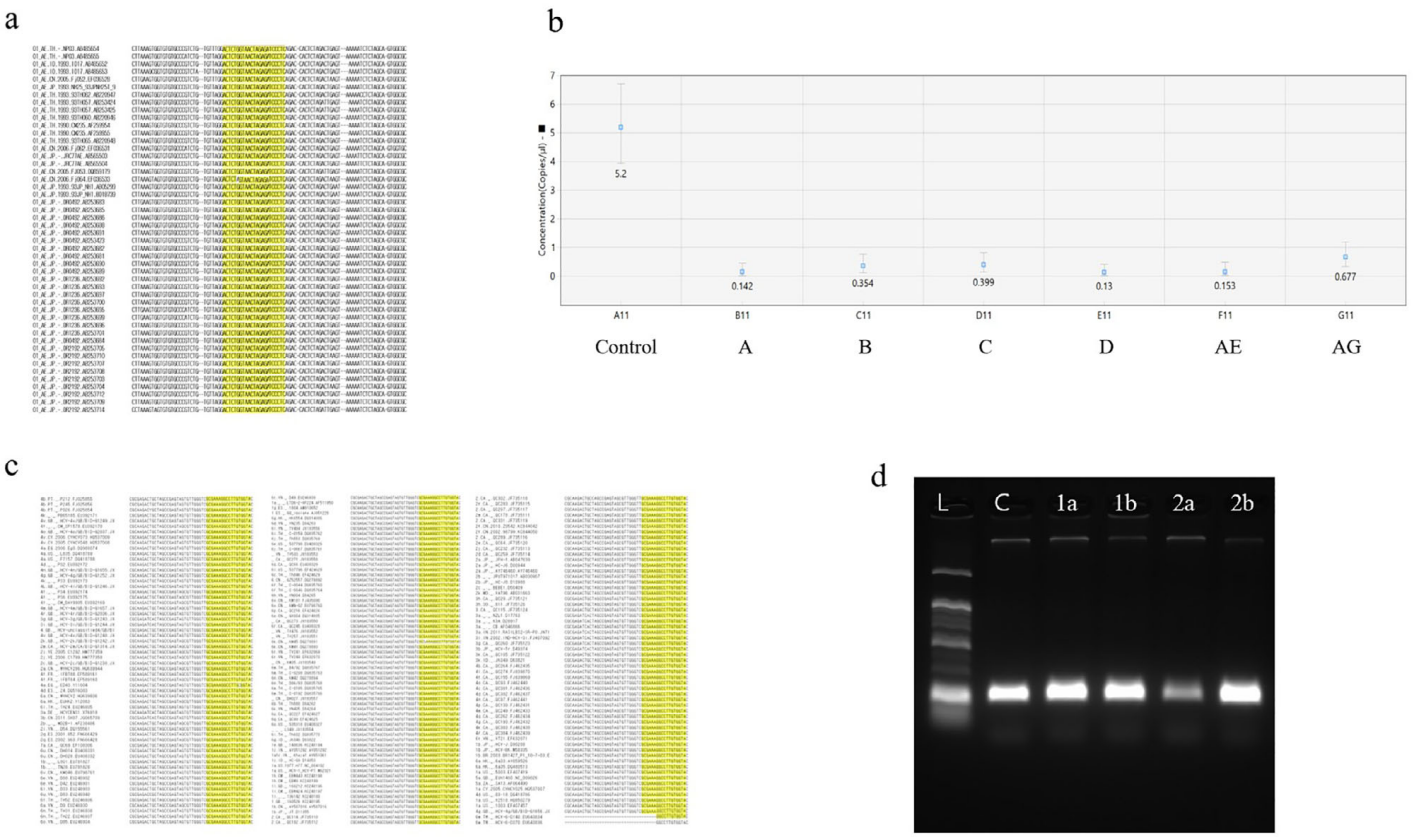
In silico alignment and experimental validation of primer and probe design for HIV‐1 and HCV. (a) Multiple sequence alignment of 840 HIV‐1 sequences representing Group M subtypes (including recombinant forms). Conserved regions selected for primer and probe design are indicated (for clarity, only a subset of the aligned sequences is shown). (b) Representative ddPCR amplification results obtained using HIV‐1 subtype reference materials, including Subtypes A–D, AE, and AG, demonstrating successful amplification across diverse subtypes. (c) Multiple sequence alignment of 201 HCV sequences spanning genotypes 1a–7a. Conserved regions used for primer and probe design are highlighted, and only representative portions of the alignment are displayed. (d) Representative agarose gel electrophoresis results confirming amplification of available HCV genotypes included in this study (L, ladder; C, control; 1a–2b, HCV genotypes 1a–2b).

Sequences were retrieved from publicly available databases, including the National Center for Biotechnology Information (NCBI), the HIV sequence database, the HCV database, and the HBV database [23, 24, 25, 26]. Sequence retrieval was performed using predefined criteria, including target genotype, genomic region, and sequence completeness. Only full‐length, non‐duplicate sequences derived from human hosts were included in the analysis. The sequence datasets and alignment results used for primer and probe design are illustrated in Figure 3. In particular, for HBV, previously published studies providing well‐curated multiple sequence alignment data were referenced and utilized to support sequence analysis [27]. Multiple sequence alignment was performed using ClustalW to identify conserved regions independent of viral subtypes, which were subsequently used for primer and probe design. For HBV, primer and probe sequences were designed based on the genomic regions targeting pgRNA as defined in a previously reported study [28].

Given the high genetic variability of HIV‐1 and HCV, primer and probe design was performed with particular attention to sequence diversity. Multiple subtype sequences were aligned, and conserved regions suitable for primer and probe binding were identified through in silico analysis. Candidate oligonucleotide sequences were designed using Primer3Plus and evaluated for specificity using NCBI basic local alignment search tool (BLAST) analysis. The in silico–designed primer and probe sets were subsequently evaluated experimentally using agarose gel electrophoresis and ddPCR to verify successful amplification across all available subtypes (Figure 3). A similar in silico design and experimental evaluation strategy was applied to HBV (data not shown).

All primers were synthesized by Macrogen (Seoul, Korea). Probes for HIV‐1, HIV‐2, HCV, and the IC, as well as the HBV probe used in the nanoplate‐based dPCR system, were synthesized by SFC (Cheongju, South Korea), whereas the HBV probe for ddPCR was synthesized by Cosmo Genetech (Seoul, Korea). The final primer and probe sets selected based on the validation process are presented in Table 1.

**TABLE 1:** Primers and probes used for the pentaplex reverse transcription droplet digital polymerase chain reaction (RT‐ddPCR) assay to detect human immunodeficiency virus (HIV)‐1, HIV‐2, hepatitis C virus (HCV), hepatitis B virus (HBV), and the internal control (IC).

Target	Gene	Primer and probe sequences	Primer/Probe concentration (nM)
HIV‐1	5′LTR	F: 5′‐ATAAAGCTTGCCTTGAGTGC‐3′ R: 5′‐GCGCCACTGCTAGAGA‐3′ P: 5′‐FAM‐ACTCTGGTAACTAGAGATCCCTC‐MGBSFCQ1‐3′	900/250
HIV‐2	5′LTR	F: 5′‐GAGAGGCTGGCAGATTGAG‐3′ R: 5′‐TTTAAGCAAGCAAGCGTGG‐3′ P: 5′‐HEX‐CGGCACTGGGCAGACGGCT‐SFCQ1‐3′	900/250
HCV	5′UTR	F: 5′‐GAACCGGTGAGTACACCG‐3′ R: 5′‐GCAAGCACCCTATCAGGC‐3′ P: 5′‐Cy5‐GACCGGGTCCTTTCTTGG‐MGBSFCQ3‐3′	900/250
HBV	*PreCore‐Core*	F: 5′‐GTGTGGATTCGCACTCCT‐3′ R: 5′‐AGGCGAGGGAGTTCTTCTT‐3′ P: 5′‐ROX‐ATGCCCCTATCTTATCAACACT‐MGB‐3′ P: 5′‐Chamel 610‐ATGCCCCTATCTTATCAACACT‐MGB‐3′a	900/250
Internal control	In‐house production	F: 5′‐CTATGCGGACCATGCGTG‐3′ R: 5′‐ATGAGACGGAGCCGTGA‐3′ P: 5′‐Cy5.5‐GCGACTCGGAGGGAGCAGGTCG‐SFCQ3‐3′	900/250 700/300^a^

^a^ This condition was experimentally optimized to ensure compatibility with the Digital LightCycler dPCR System, including adjustment of reaction conditions and substitution of fluorescent dyes where required, while maintaining the original probe sequence.

Subsequently, primer and probe concentrations for the pentaplex reaction were optimized with reference to the corresponding singleplex assay results to ensure consistent analytical performance in a high‐order pentaplex environment. Changes in cluster patterns and the presence of rain effects were evaluated through comparative analysis of one‐dimensional (1D) and two‐dimensional (2D) fluorescence amplitude plots. Quantitative performance was assessed by comparing quantified concentrations (copies/reaction) between singleplex and pentaplex assays using identical template inputs, and statistical differences were evaluated using the Mann–Whitney *U* test.

To further assess multiplex‐induced effects, fluorescence amplitude distributions were analyzed using both 1D and 2D cluster plots generated from ddPCR data. These analyses were performed to evaluate cluster integrity and potential multiplex‐induced artifacts in pentaplex reactions compared to singleplex controls.

### 2.5 Pentaplex RT‐ddPCR

ddPCR analysis was performed using the QX600 ddPCR system (Bio‐Rad, Hercules, CA, USA) with the ddPCR Multiplex Supermix (Bio‐Rad), according to the manufacturer's instructions. The reaction mixture consisted of 5.5 µL of ddPCR Multiplex Supermix, 0.29 µL of DTT, 900 nM each of forward and reverse primers for each target, 250 nM of each probe, cDNA templates, and ultrapure water (iNtRON, Gyeonggi, South Korea), adjusted to a final volume of 22 µL, of which 20 µL was emulsified with 70 µL of droplet‐generating oil (Bio‐Rad) using a QX200 Droplet Generator (Bio‐Rad) to partition each sample into droplets.

The emulsified samples were transferred to a 96‐well plate and sealed with foil using a PX1 PCR Plate Sealer (Bio‐Rad). Thermal cycling was performed in a T100 Thermocycler (Bio‐Rad) under the following conditions: 95°C for 10 min; 50 cycles of 94°C for 30 s and 59°C for 60 s; followed by 98°C for 10 min, and a final hold at 4°C. The ramp rate was set to 2°C/s. Droplets were subsequently read using the QX600 ddPCR system reader (Bio‐Rad), and data were analyzed using QX Manager Software version 2.2 (Standard Edition; Bio‐Rad).

### 2.6 Assay Validation of the Pentaplex RT‐ddPCR

The analytical performance of the pentaplex RT‐ddPCR assay was systematically evaluated in terms of linearity, specificity, lower limit of quantification (LLoQ), limit of detection (LoD), cut‐off determination, robustness, precision, and reproducibility.

Linearity was assessed using HIV‐1, HIV‐2, HCV, and HBV reference materials obtained from NIBSC, together with internally synthesized IC RNA. All targets were combined in a single tube, extracted, and reverse‐transcribed into cDNA. Five serial two‐fold dilutions were prepared from the resulting cDNA stock and analyzed simultaneously using the pentaplex RT‐ddPCR assay. Each experiment was performed in triplicate, and linearity was evaluated on the basis of mean values.

Specificity was evaluated by separately extracting and reverse‐transcribing HIV‐1, HIV‐2, HCV, HBV, and IC RNAs. Each target was analyzed individually using the pentaplex RT‐ddPCR assay, and target specificity was assessed using 1D amplitude plots to verify distinct signal separation without cross‐reactivity.

The LLoQ was determined using combined nucleic acid targets prepared in a single tube, followed by five serial two‐fold dilutions. Each dilution was tested in eight replicates, and the LLoQ was defined as the lowest concentration with coefficient of variation (CV) <25%, together with consideration of the standard deviation (SD). The same dataset was further analyzed to estimate the 95% LoD using probit regression analysis, which defined the lowest concentration at which 95% of positive reactions were detected.

For comparative evaluation, the performance of the pentaplex RT‐ddPCR assay at the LoD was compared with that of a commercial RT‐qPCR kit (HCV/HBV/HIV‐1/HIV‐2 Real‐TM; Sacace Biotechnologies S.r.l., Como, Italy). Although the commercial kit does not support simultaneous detection of all five targets in a single reaction, it allows multiplex detection of three targets per tube, namely, HIV‐1/HIV‐2/IC or HCV/HBV/IC. Accordingly, identical samples used for LoD determination were analyzed in separate reactions following the manufacturer's instructions to enable a direct performance comparison for each viral target. Comparability between the two assays, and the trueness of the pentaplex RT‐ddPCR assay, were indirectly assessed by examining the overlap of the 95% confidence intervals (CIs) of the estimated LoD values for each viral target.

The cut‐off value of the pentaplex assay was determined based on sensitivity and specificity analysis. The cut‐off was defined as the lowest concentration at which no false‐positive or false‐negative results were observed, using the same dataset applied for LLoQ and LoD analyses [29].

(1) $$\mathrm{Sensitivity}=\frac{\mathrm{True}\;\mathrm{positive}\;\left(\mathrm{TP}\right)}{\mathrm{True}\;\mathrm{positive}\;\left(\mathrm{TP}\right)+\mathrm{False}\;\mathrm{negative}\;\left(\mathrm{FN}\right)}\;$$

(2) $$\mathrm{Specificity}=\frac{\mathrm{True}\;\mathrm{negative}\;\left(\mathrm{TN}\right)}{\mathrm{True}\;\mathrm{negative}\;\left(\mathrm{TN}\right)+\mathrm{False}\;\mathrm{positive}\;\left(\mathrm{FP}\right)}$$

Robustness was evaluated by assessing the impact of thermal cycler type and the time interval between droplet generation and PCR amplification. Results obtained using two different thermal cyclers and different incubation times prior to PCR were compared, and variability was quantified using CV.

Precision was assessed by alternately analyzing high‐concentration samples and negative plasma samples spiked with the IC. Precision was evaluated based on CV values for the viral targets in high‐concentration samples and by monitoring false positives and IC variability in negative samples. Standard error was additionally calculated to assess the reliability of the measured means.

Reproducibility was evaluated through cross‐platform comparison using droplet‐based and nanowell‐based dPCR systems. The pentaplex assay was applied to the Digital LightCycler dPCR System, and absolute quantification results from both platforms were compared. For the Digital LightCycler dPCR System, assay conditions were independently optimized in advance, including annealing temperature optimization and clarification of positive partition clustering (Table S2 in Supporting Information Data 1). Following this platform‐specific optimization, reproducibility was assessed based on CV values and statistical comparison of quantification results.

### 2.7 Evaluation of the Pentaplex RT‐ddPCR Assay Using Externally Sourced HIV‐1 Materials

Externally sourced pathogen resources were subjected to nucleic acid extraction from 140 µL of input material. Following extraction, DNase treatment and cDNA synthesis were performed as described above. For ddPCR analysis, 5 µL of the processed nucleic acid (post‐DNase treatment and cDNA synthesis) was used as input per reaction. Assay performance was evaluated based on qualitative concordance with results obtained from external antibody testing and nucleic acid testing performed by the supplying institution. The experimental conditions used by the external institution are described in Table S3 Supporting Information Data 1. In addition, quantitative results were converted from copies/µL to the internationally accepted unit of copies/mL to facilitate visual comparison, and the conversion formula was based on previously reported methods [30].

### 2.8 Statistical Analysis

The threshold for ddPCR analysis was calculated using QX Manager Software version 2.2 (Standard Edition; Bio‐Rad). Reproducibility assessment for the Roche dPCR system was performed using Digital LightCycler development software version 1.1 (Roche). All other statistical analyses were performed using R version 4.3.3 (R Foundation for Statistical Computing, Vienna, Austria).

### 2.9 Use of Artificial Intelligence (AI)

AI‐assisted tools (ChatGPT, OpenAI, USA) were used solely for language editing and English translation of the manuscript. All outputs generated by the AI tool were carefully reviewed, cross‐checked against the original data and references, and revised by the authors to ensure scientific accuracy and integrity. The authors take full responsibility for all content presented in this manuscript.

## 3 Results

### 3.1 Optimization of Concentration of Primers and Probes

**FIGURE 4:**
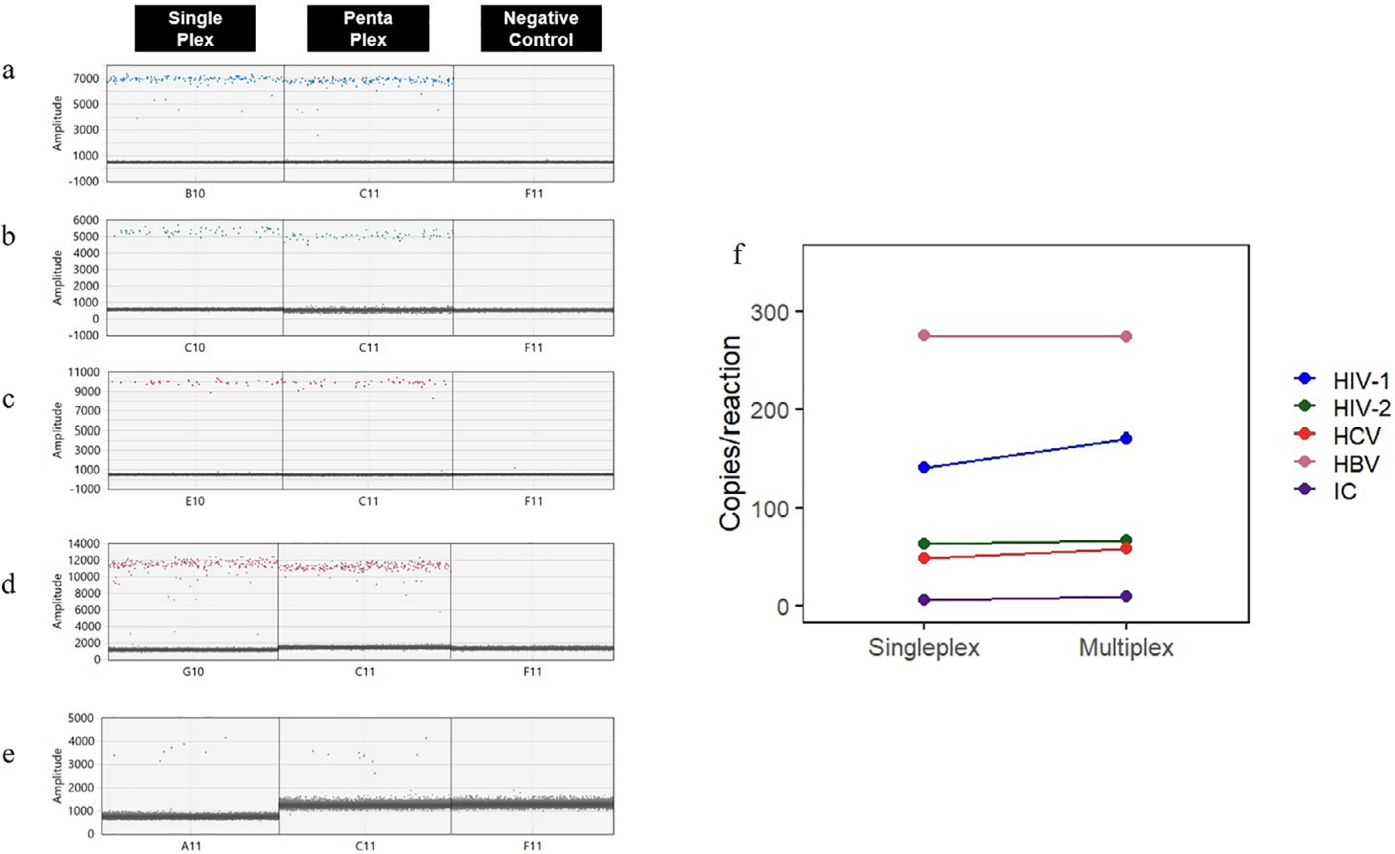
Comparison of singleplex and pentaplex ddPCR assays. Quantified copies/µL of each viral target were measured using both singleplex and pentaplex ddPCR to evaluate the effect of multiplexing. Parts (a–e) show fluorescence amplitude plots for each channel: FAM (HIV‐1), HEX (HIV‐2), Cy5 (HCV), ROX (HBV), and Cy5.5 (IC), respectively. Part (f) presents the statistical comparison between singleplex and pentaplex conditions using the Mann–Whitney *U* test. HBV, hepatitis B virus; HCV, hepatitis C virus; HIV, human immunodeficiency virus; IC, internal control.

A comparison between singleplex and pentaplex ddPCR assays was performed by measuring copies for each viral target (Figure 4). Analysis of the 1D fluorescence amplitude plots revealed no appreciable increase in rain droplets or shifts in cluster positions under pentaplex conditions compared to singleplex reactions, indicating that droplet partitioning and signal separation were well maintained in the multiplex environment (Figure 4a–e). For the Cy5.5 channel used for IC detection, a slight upward shift in the negative cluster was observed under pentaplex conditions. However, as this channel is intended for qualitative detection rather than quantitative analysis, this change had minimal impact on assay performance. Moreover, the observed variation did not interfere with threshold setting, and clear discrimination between positive and negative droplets was preserved.

Statistical comparison using the Mann–Whitney *U* test showed that some targets exhibited significantly higher quantified concentrations under pentaplex conditions, whereas the remaining targets showed no statistically significant differences. In particular, although HBV showed a slight decrease in mean quantified values under pentaplex conditions, this difference was not statistically significant (*p* > 0.05). These results indicate that multiplexing did not adversely affect quantitative performance (Figure 4f).

**FIGURE 5:**
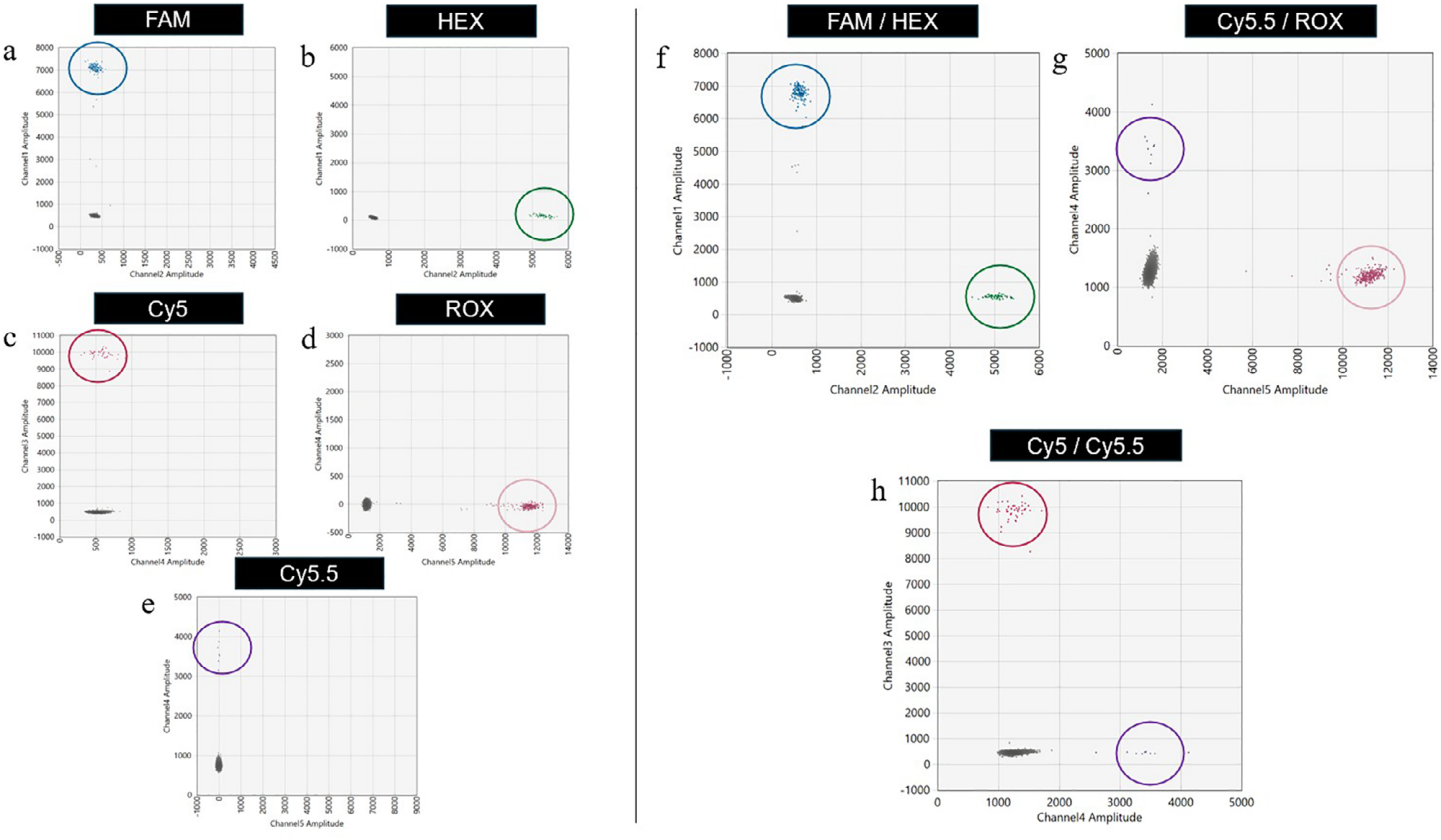
Two‐dimensional (2D) fluorescence amplitude plots of singleplex and pentaplex ddPCR assays. Parts (a–e) show dual‐channel 2D plots for singleplex reactions, in which only a single target was amplified in each channel (FAM, HEX, Cy5, ROX, and Cy5.5). Parts (f–h) present representative dual‐channel combinations (FAM/HEX, Cy5.5/ROX, and Cy5/Cy5.5) under pentaplex conditions. Across all channel combinations, no noticeable shifts in cluster positions were observed, and clear separation between positive and negative clusters was maintained under pentaplex conditions.

Analysis of the 2D fluorescence amplitude plots further confirmed the consistency between singleplex and pentaplex conditions (Figure 5). No noticeable shifts in cluster positions were observed across all channel combinations, and well‐defined cluster separation was maintained in the multiplex environment. These results indicate that multiplexing did not introduce signal interference or compromise cluster resolution.

### 3.2 Linearity of the Pentaplex RT‐ddPCR

**FIGURE 6:**
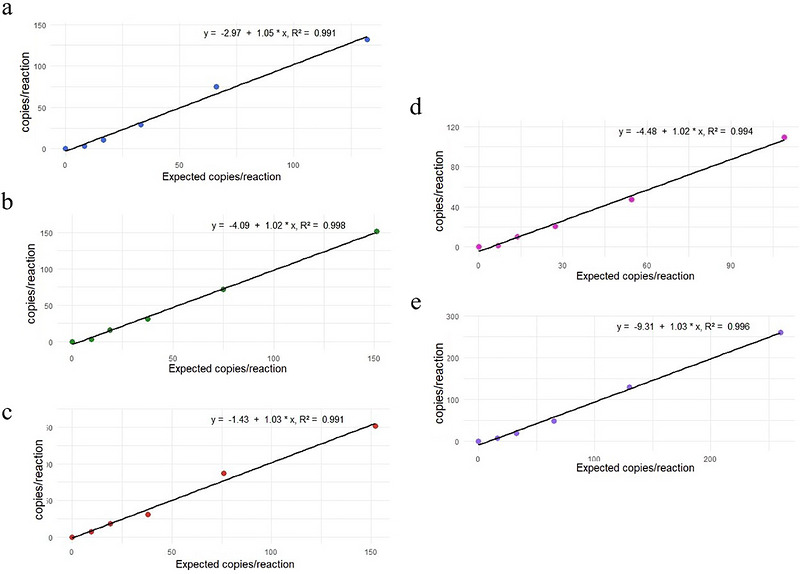
Linearity of the pentaplex RT‐ddPCR (reverse transcription droplet digital PCR) assay for HIV‐1, HIV‐2, HCV, HBV, and IC. (a–e) Scatter plots with *R* ^2^ showing the relationship between expected copies per 20 µL and measured ddPCR results per 20 µL for (a) HIV‐1, (b) HIV‐2, (c) HCV, (d) HBV, and (e) IC.

The linearity of the pentaplex RT‐ddPCR assay was evaluated using five concentration points generated by serial two‐fold dilutions of the cDNA templates. The results were expressed as copies/µL and copies/20 µL, which corresponds to copies/reaction. The expected copies/reaction were calculated by halving the initial concentration at each dilution step. Linear regression analysis demonstrated high coefficients of determination (*R* ^2^) for HIV‐1, HIV‐2, HCV, HBV, and the IC, with values of 0.991, 0.998, 0.991, 0.994, and 0.996, respectively, indicating a strong linear relationship across the tested concentration range (Figure 6). Linearity assessment in the pentaplex format showed that all targets achieved *R* ^2^ values greater than 0.99, suggesting the absence of significant cross‐interference among targets within the multiplex reaction.

### 3.3 Specificity of the Pentaplex RT‐ddPCR

**FIGURE 7:**
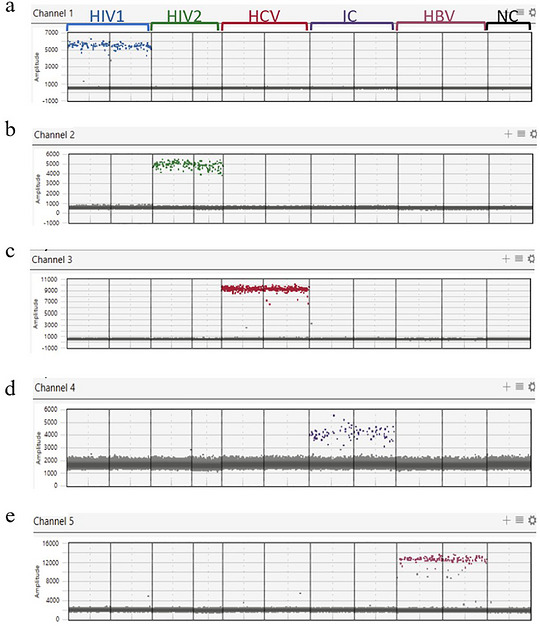
Specificity of the pentaplex RT‐ddPCR (reverse transcription droplet digital PCR) assay was demonstrated using one‐dimensional (1D) amplitude plots (a–e). Representative 1D plots showing specific amplification of (a) HIV‐1, (b) HIV‐2, (c) HCV, (d) HBV, and (e) IC. The negative control (NC) showed no nonspecific amplification. HBV, hepatitis B virus; HCV, hepatitis C virus; HIV, human immunodeficiency virus; IC, internal control.

Despite the use of a common master mix containing primers and probes for all targets, positive clusters were observed only in wells in which the corresponding target virus was dispensed. No positive amplification was detected in the wells in which distilled water was used as the negative control (NC). Notably, all viral samples contained human RNA; however, only target‐specific signals were detected without nonspecific amplification. These findings indicate that the pentaplex RT‐ddPCR assay maintains high specificity even within complex matrices, such as human‐derived RNA (Figure 7).

### 3.4 LLoQ of the Pentaplex RT‐ddPCR

**FIGURE 8:**
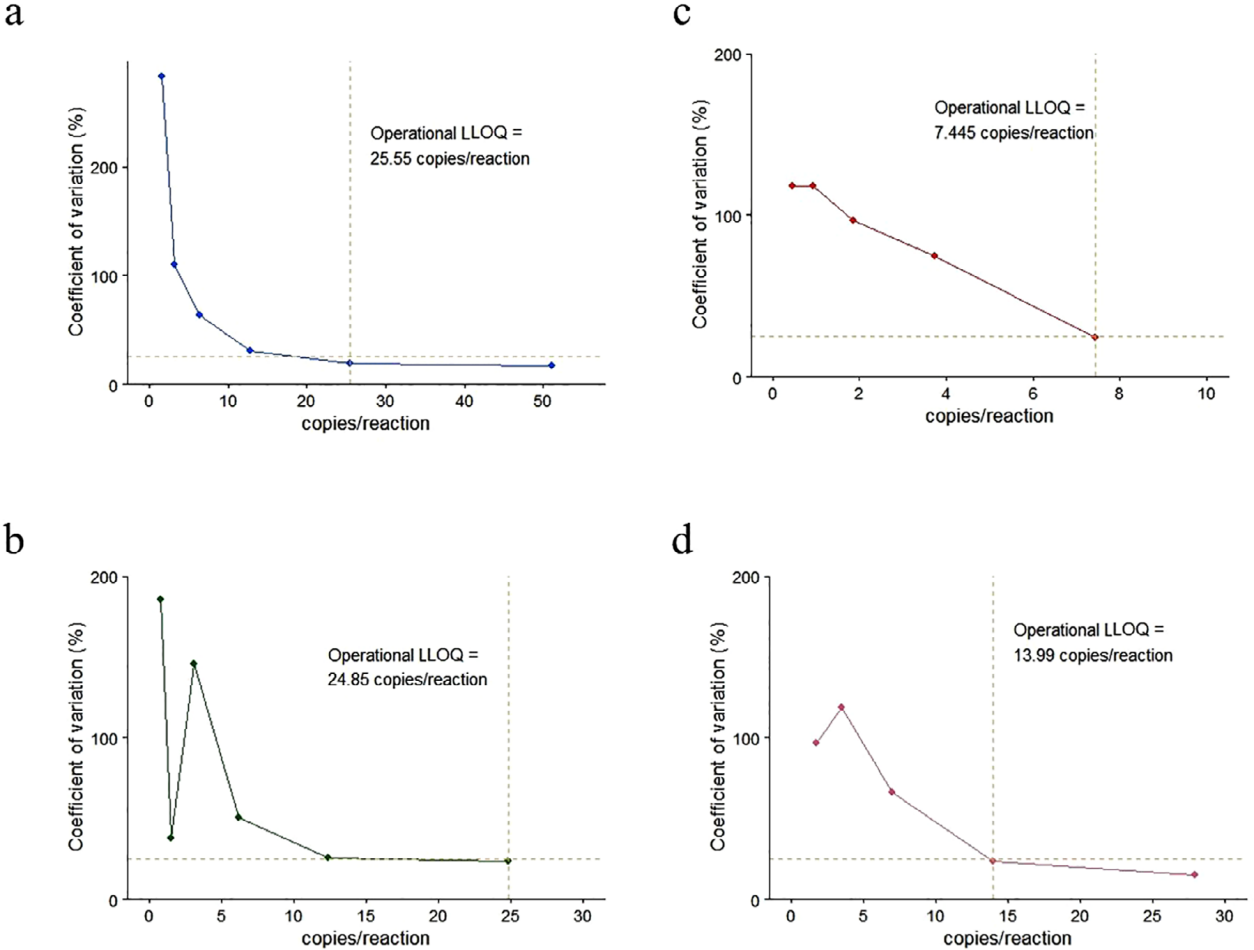
The LLoQ of the pentaplex RT‐ddPCR assay. Expected concentrations represent nominal input concentrations calculated from serial dilutions. Measured mean values indicate the average copies per reaction obtained from eight replicate measurements. The LLoQ for each target was defined as the lowest concentration with a coefficient of variation (CV) ≤25%. Panels represent (a) HIV‐1, (b) HIV‐2, (c) HCV, and (d) HBV. LLoQ, lower limit of quantification.

The LLoQ for the pentaplex RT‐ddPCR assay was determined by testing serially diluted samples simultaneously containing HIV‐1, HIV‐2, HCV, HBV, and IC. Six low‐concentration samples were prepared by two‐fold serial dilution and measured in eight replicates (Table S1 in the Supporting Information Data 2). Expected copies/reactions were calculated by halving the initial concentration at each dilution step. The LLoQ was defined as the concentration at which the CV across the eight replicates did not exceed 25%. The determined LLoQ values for HIV‐1, HIV‐2, HCV, and HBV were 25.5 copies/reaction, 24.8 copies/reaction, 7.4 copies/reaction, and 27.9 copies/reaction, respectively (Figure 8). For HCV, although the LLoQ was determined at the highest tested concentration within the evaluated range, the progressively decreasing CV values observed across subsequent lower concentrations exhibited a clear trend, supporting the suitability of this concentration as the operational LLoQ.

### 3.5 LoD Determination of the Pentaplex RT‐ddPCR Assay and Comparison With RT‐qPCR

**FIGURE 9:**
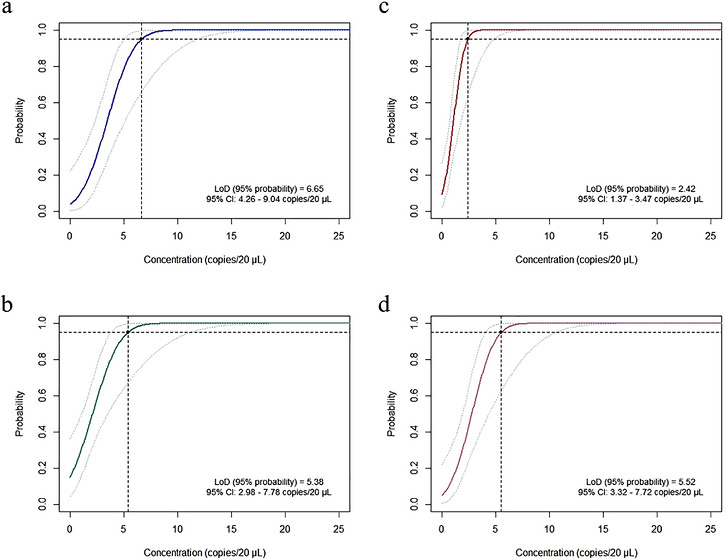
The LoD of the pentaplex RT‐ddPCR (reverse transcription droplet digital PCR) assay was determined using a probit regression model at the 95% confidence level. Each concentration was tested in eight replicates to evaluate assay sensitivity. Panels represent (a) HIV‐1, (b) HIV‐2, (c) HCV, and (d) HBV. CI, confidence interval; LoD, limit of detection.

As described in Section 3.3, the number of expected copies per reaction was calculated by halving the initial concentration at each dilution step (Table S2 in the Supporting Information Data 2). On the basis of 95% probit regression analysis, the LoD values of the pentaplex RT‐ddPCR assay were determined to be 6.65 copies/reaction (95% CI: 4.26–9.04 copies/reaction) for HIV‐1, 5.38 copies/reaction (95% CI: 2.96–7.78 copies/reaction) for HIV‐2, 2.42 copies/reaction (95% CI: 1.37–3.47 copies/reaction) for HCV, and 5.52 copies/reaction (95% CI: 3.32–7.72 copies/reaction) for HBV (Figure 9).

**FIGURE 10:**
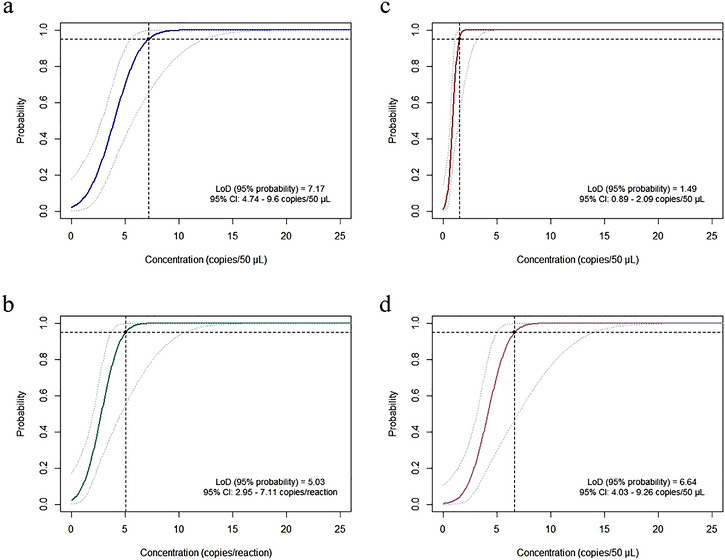
The LoD of the commercial RT‐qPCR (reverse transcription quantitative PCR) kit was determined using a probit regression model at the 95% confidence level. Each concentration was tested in eight replicates to evaluate assay sensitivity. Panels represent (a) HIV‐1, (b) HIV‐2, (c) HCV, and (d) HBV. CI, confidence interval; LoD, limit of detection.

To evaluate the relative analytical sensitivity of the pentaplex RT‐ddPCR assay, the same dilution series was subsequently analyzed using a commercial RT‐qPCR kit. The LoD values obtained with the RT‐qPCR assay were 7.17 copies/reaction (95% CI: 4.74–9.6 copies/reaction) for HIV‐1, 5.03 copies/reaction (95% CI: 2.95–7.11 copies/reaction) for HIV‐2, 1.49 copies/reaction (95% CI: 0.89–2.09 copies/reaction) for HCV, and 6.64 copies/reaction (95% CI: 4.03–9.26 copies/reaction) for HBV (Figure 10).

**FIGURE 11:**
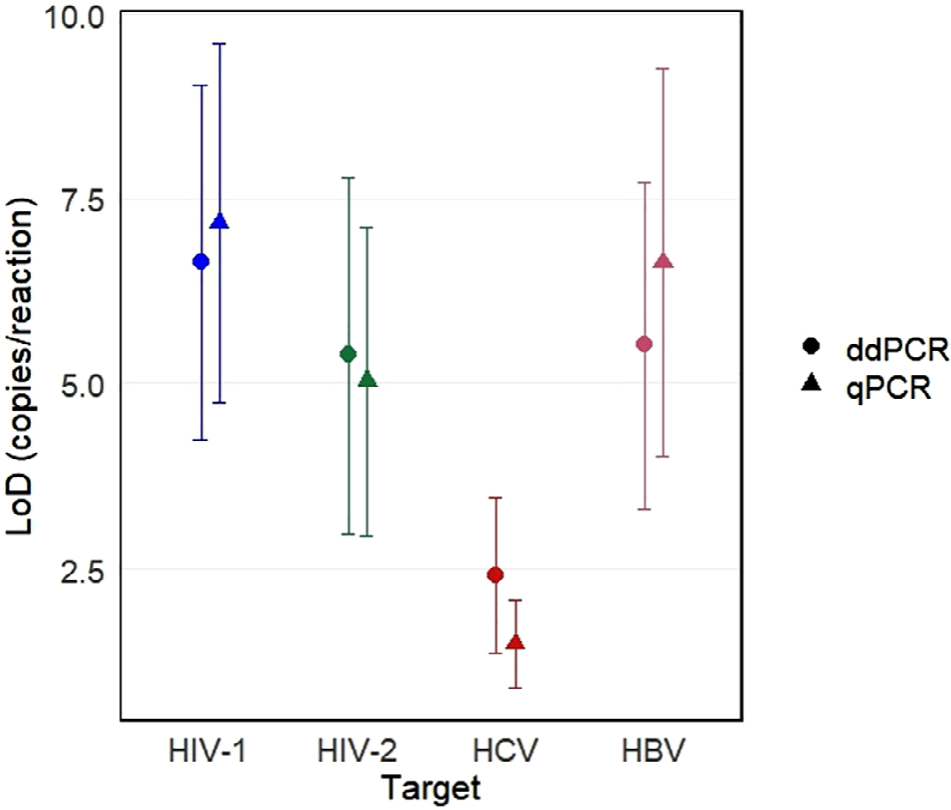
Comparison of the LoD between droplet digital PCR and quantitative PCR for HIV‐1, HIV‐2, HCV, and HBV. LoD values were estimated using a 95% probit regression model. Points indicate the estimated LoD values (copies/reaction), and error bars represent the corresponding 95% CI. Identical samples used for LoD determination were analyzed using both ddPCR and a commercial RT‐qPCR kit according to the manufacturer's instructions. CI, confidence interval; ddPCR, droplet digital polymerase chain reaction; HBV, hepatitis B virus; HCV, hepatitis C virus; HIV, human immunodeficiency virus; LoD, limit of detection; qPCR, quantitative polymerase chain reaction.

A comparative evaluation of the LoD values obtained from the RT‐ddPCR and RT‐qPCR assays was performed by examining the overlap of the 95% CI for each viral target. As shown in Figure 11, the CI of the two methods overlapped for all targets, including HIV‐1, HIV‐2, HCV, and HBV, indicating no apparent difference in analytical sensitivity between the assays. The absence of systematic differences between the two methods further suggests acceptable trueness of the pentaplex RT‐ddPCR assay when indirectly assessed through comparison with a commercially validated RT‐qPCR assay. Notably, although the pentaplex RT‐ddPCR assay enables the simultaneous detection of five targets in a single reaction, the commercial RT‐qPCR kit employs a triplex configuration, allowing the detection of three targets per reaction. Despite this difference in multiplexing capacity, comparable LoD performance was observed across all viral targets. Collectively, this overlap‐based assessment supports the conclusion that the analytical sensitivity and trueness of the pentaplex RT‐ddPCR assay are comparable to those of the commercial RT‐qPCR kit, while offering increased multiplexing capability.

### 3.6 Cut‐Off Value of the Pentaplex RT‐ddPCR

**FIGURE 12:**
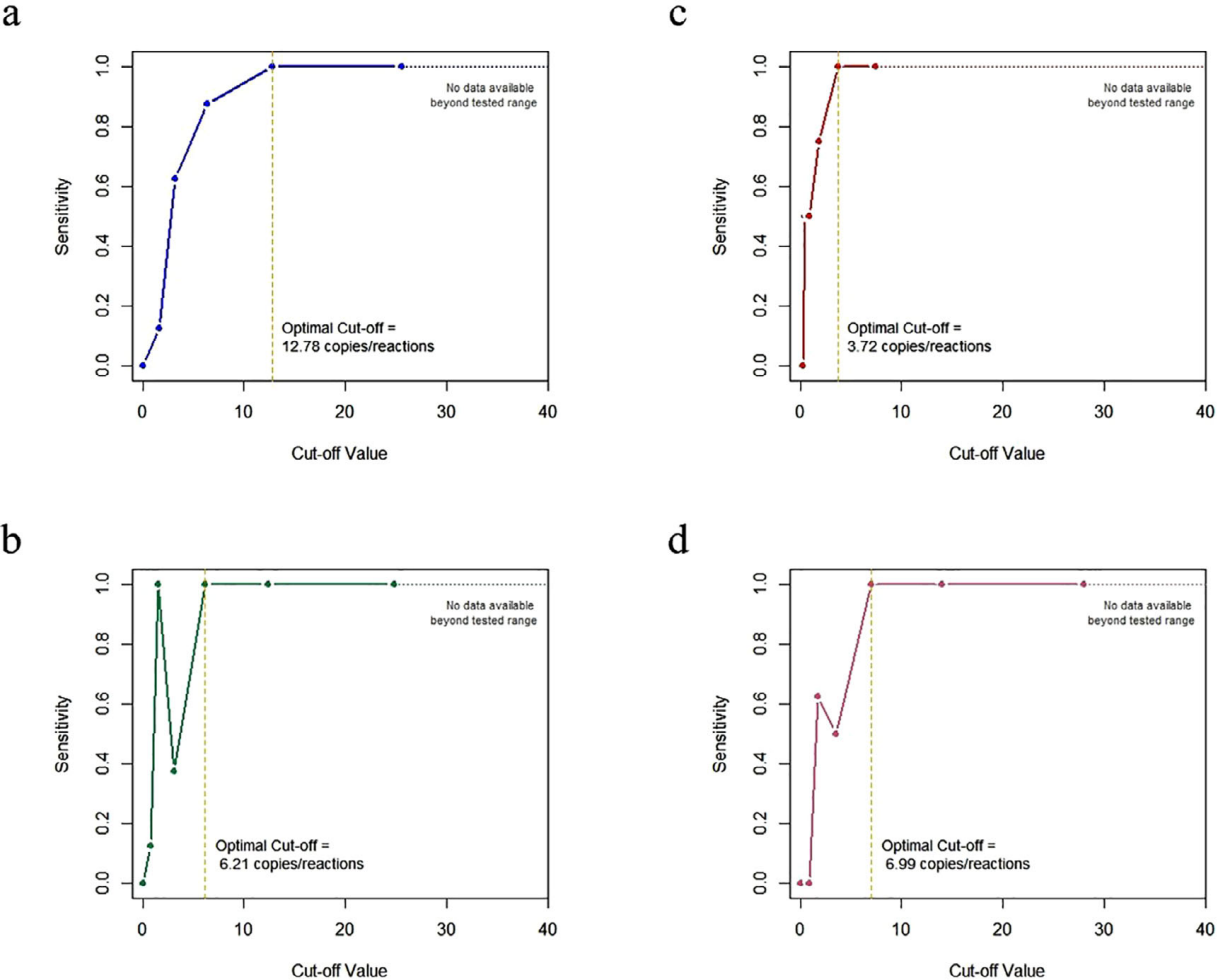
Determination of optimal cut‐off values based on the Youden index. Sensitivity curves were generated across a range of cut‐off values for each viral target, and the optimal cut‐off was defined as the point at which the Youden index (sensitivity + specificity − 1) reached its maximum value. Vertical dashed lines indicate the selected optimal cut‐off values (copies/reaction). Panels represent (a) HIV‐1, (b) HIV‐2, (c) HCV, and (d) HBV.

The cut‐off value was set at the lowest concentration with no false positives or negatives, corresponding to a Youden's index of 1. The cut‐off values for each target were determined as follows: 12.78 copies/reaction for HIV‐1, 6.21 copies/reaction for HIV‐2, 3.72 copies/reaction for HCV, and 6.99 copies/reaction for HBV (Figure 12).

### 3.7 Reliability of the Pentaplex RT‐ddPCR Assay

To comprehensively evaluate the analytical reliability of the pentaplex RT‐ddPCR assay, its robustness, precision, and reproducibility were systematically assessed under varying experimental conditions. These validation parameters were investigated to confirm that the assay maintains consistent quantitative performance despite changes in instrumentation, workflow timing, and dPCR platforms (Tables S3–S5 in the Supporting Information Data 2).

**FIGURE 13:**
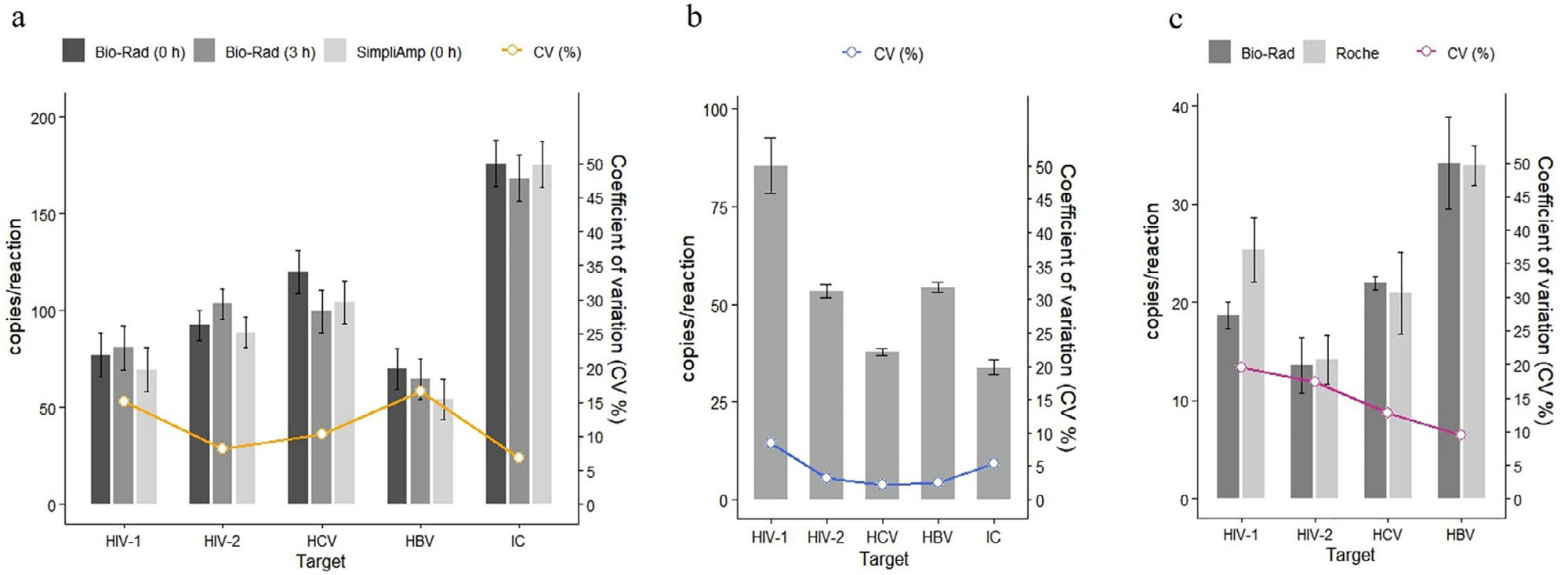
Performance evaluation of the pentaplex RT‐ddPCR (reverse transcription droplet digital PCR) assay: (a) robustness across different reaction conditions (Bio‐Rad 0 h, Bio‐Rad 3 h, and SimpliAmp 0 h); (b) precision of viral target quantification; and (c) comparison between Bio‐Rad and Roche platforms. Bars represent mean copies per reaction, error bars indicate SD, and lines denote CV. CV, coefficient of variation; HBV, hepatitis B virus; HCV, hepatitis C virus; HIV, human immunodeficiency virus; IC, internal control; SD, standard deviation.

First, assay robustness was evaluated by examining whether key experimental variables influenced the quantitative results. The tested variables included the use of two different thermal cyclers (T100 and SimpliAmp) and the timing of PCR amplification (immediately after droplet generation versus 3 h post‐generation). For all five targets, the observed CV remained below 15%, with values of 7.57% for HIV‐1, 8.04% for HIV‐2, 10.00% for HCV, 12.64% for HBV, and 2.47% for the IC, indicating that these variables had minimal impact on assay performance (Figure 13a).

The precision of the assay was subsequently assessed by alternately distributing high‐concentration samples and NCs to evaluate repeatability under the same analytical conditions. The CV values for HIV‐1, HIV‐2, HCV, and HBV in the high‐concentration samples were 8.39%, 3.26%, 2.13%, and 2.50%, respectively, confirming high measurement consistency. No viral targets were detected in the NC samples, demonstrating the absence of false‐positive signals, whereas the IC was consistently detected, indicating no false negatives. The CV of the IC copies/reaction in duplicate NCs was 5.42%, further confirming that the presence of high‐concentration samples did not affect IC quantification (Figure 13b).

Finally, assay reproducibility was evaluated by applying the same analytical protocol to both droplet‐based and nanowell‐based dPCR platforms. Reproducibility was assessed based on two criteria: maintenance of CV values below 20% at the previously determined LLoQ and the absence of statistically significant differences between platforms. Using mean values from three independent replicates, the CVs for HIV‐1, HIV‐2, HCV, HBV, and IC were 17.95%, 17.38%, 12.76%, 9.46%, and 8.79%, respectively. In addition, no statistically significant differences were observed between the two platforms for any target across all tested concentrations (all *p* values >0.1), demonstrating high inter‐platform reproducibility of the assay (Figure 13c).

### 3.8 Applicability of the Pentaplex RT‐ddPCR Assay to Externally Sourced HIV‐1 Materials

All externally sourced pathogen resources yielded detectable amplification signals for the corresponding viral targets, demonstrating the applicability of the pentaplex RT‐ddPCR assay beyond international reference materials. Because substantial differences existed between the external RT‐qPCR workflow and the pentaplex RT‐ddPCR assay in terms of infected plasma input and template input amounts, the evaluation was focused on qualitative concordance rather than direct quantitative comparison (Table S4 in the Supporting Information Data 1). In addition, despite being human‐derived matrices closely resembling clinical specimens as described above, the externally sourced pathogen resources were correctly classified by the pentaplex RT‐ddPCR assay, showing complete qualitative concordance with the external RT‐qPCR results (Table 2). No false‐positive or false‐negative results were observed among the 12 positive samples and 3 NCs. Furthermore, no amplification signals were detected in the non‐target channels, and the IC was successfully detected in all reactions. These results indicate that the assay maintained target‐specific detection performance even in human‐derived plasma matrices.

**TABLE 2:** Concordance of pentaplex reverse transcription droplet digital polymerase chain reaction (RT‐ddPCR) and external reverse transcription quantitative polymerase chain reaction (RT‐qPCR) results for externally sourced human immunodeficiency virus (HIV)‐1 materials.

RT‐qPCR result	ddPCR negative	ddPCR positive	Total
**Negative**	3	0	3
**Positive**	0	12	12
**Total**	3	12	15

## 4 Discussion

The pentaplex RT‐ddPCR assay, presented in this study, is a ddPCR‐based multiplex analytical system capable of simultaneously detecting HIV‐1, HIV‐2, HCV, and HBV, as well as an IC. Commercial diagnostic kits are typically based on single‐target qPCR systems, which limit their multiplexing capacity. In contrast, screening kits often allow multiplex configurations but are limited in their ability to perform accurate quantitative analyses. This assay was specifically designed to enable both qualitative and quantitative analyses by leveraging the absolute quantification capability inherent to ddPCR. Compared to conventional methods based on relative quantification, this approach provides more accurate and consistent quantification. Notably, the four viral targets included in this assay have been designated by the WHO as mandatory testing items for blood‐based reference materials, highlighting the practical relevance of establishing a unified assay system that encompasses all required pathogens.

The analytical performance of the developed assay was further evaluated through comparison with a commercial research‐use‐only (RUO) kit targeting the same viruses. The commercial kit detects HIV‐1, HIV‐2, and IC or HCV, HBV, and IC in separate triplex configurations, whereas the assay presented in this study enables simultaneous detection of all five targets in a single pentaplex format. Despite the higher level of multiplexing, the limits of detection obtained using the two assays were comparable, as demonstrated by the consistent overlap of the 95% CI across all viral targets. This result indicates that the developed pentaplex RT‐ddPCR assay achieves analytical sensitivity comparable to that of existing commercial methods while offering improved efficiency and cost‐effectiveness through simultaneous multi‐target detection.

Although clinical specimens were not included in this study, externally sourced pathogen resources obtained from an independent institution were evaluated as an alternative approach to partially address this limitation. These materials consisted of human plasma–derived HIV‐1–positive samples, closely resembling clinical specimens. All externally sourced HIV‐1–derived materials were successfully detected and quantified using the pentaplex RT‐ddPCR assay, showing complete qualitative concordance with external IVD (in vitro diagnostics) RT‐qPCR results despite differences in input amounts, demonstrating that the assay is applicable beyond in‐house reference materials and performs reliably on independently sourced materials. This finding supports the robustness of the assay design, particularly given the high genetic diversity characteristic of HIV‐1.

Nevertheless, this study had several limitations. First, the number of samples analyzed was limited, which constrained the scope of statistical analysis. In addition, although samples resembling clinical specimens were included, the evaluation was restricted to HIV‐1, and the absence of actual clinical specimens limited the ability to fully assess real‐world clinical applicability. Second, although comparative analyses were performed using both a commercial RUO RT‐qPCR kit and an IVD RT‐qPCR kit, the IVD assay evaluated was based on a singleplex format. A direct comparison with multiplex, fully automated diagnostic platforms widely used as clinical standards worldwide was not conducted.

Furthermore, the IC used in this study consisted of a synthetic naked RNA molecule. Although this IC was effective for identifying false‐negative results, it had limitations in fully reflecting the efficiency of nucleic acid extraction and the overall pre‐analytical process from intact viral particles. Although armored RNA or pseudotyped virus–based controls may be more appropriate in this regard, a synthetic RNA‐based IC was adopted in this study to prioritize the evaluation of analytical performance and quantitative characteristics of the multiplex detection system.

Future studies incorporating larger sample sizes and clinical specimens, along with comparative evaluation against automated clinical diagnostic platforms, will be necessary to further establish the clinical utility of the proposed assay.

Despite these limitations, this study presents, to our knowledge, the first ddPCR‐based pentaplex system capable of simultaneously detecting and quantifying HIV‐1, HIV‐2, HCV, and HBV nucleic acids. Validation of the assay was conducted in accordance with the principles outlined in ISO 20395:2019, underscoring both its scientific robustness and its potential practical value.

## 5 Conclusion

In this study, a pentaplex RT‐ddPCR assay capable of simultaneously detecting HIV‐1, HIV‐2, HCV, HBV, and an IC was developed. This platform enabled integrated qualitative detection and absolute quantification within a single multiplex assay.

The proposed assay demonstrates potential applicability for a wide range of purposes, including diagnostics, screening, and reference material validation, and its analytical performance was validated in accordance with the principles outlined in ISO 20395:2019. Furthermore, successful implementation on both droplet‐based and nanowell plate–based dPCR platforms confirmed its reproducibility across dPCR technologies.

Although this study has certain limitations, including a limited number of test samples and the absence of direct comparisons with fully automated multiplex clinical diagnostic platforms, the applicability of the assay was demonstrated through evaluation using HIV‐1–derived materials obtained from an external institution under conditions resembling clinical specimens. Collectively, these findings highlight the potential of the proposed assay as a versatile tool for future clinical diagnostics and laboratory quality control applications.

## Author Contributions

**Soo Yeon Lim**: writing – original draft, validation, visualization, and methodology. **Un Na Koh**: methodology, formal analysis, and project administration. **Ah Leum Kim**: investigation and writing – review and editing. **Yebin Kim**: investigation and validation. **Ga Eun Kim**: investigation and writing – review and editing. **Si‐Keun Lim**: conceptualization, supervision and funding acquisition.

## Ethics Statement

The authors have nothing to report.

## Consent

The authors have nothing to report.

## Conflicts of Interest

The authors declare the following competing interests. A patent application related to the subject matter of this manuscript has been filed by the authors. The patent, titled “Kit for simultaneous detection or quantification of HIV‐1, HIV‐2, HCV and HBV capable of determining false negative,” was filed on August 13, 2025, under application number 10‐2025‐0111973 (receipt number 1‐1‐2025‐0921245‐37). The applicant is Sungkyunkwan University Industry–Academic Cooperation Foundation.

## Supporting information




**Supporting File 1**: elps70098‐sup‐0001‐SupMat‐Data‐1.docx.

**Supporting File 2**: elps70098‐sup‐0002‐SupMat‐Data‐2.xlsx.

## Data Availability

All data generated or analyzed during this study are included in this published article and its Supporting Information Data.
