# Supplementary material for: Development of Pentaplex Reverse Transcription Droplet Digital PCR Assay for Simultaneous Detection and Absolute Quantification of HIV‐1, HIV‐2, HCV, and HBV With Internal Control
Source: Electrophoresis. 2026 May 27;47(7):629–43. doi: 10.1002/elps.70098 (PMC13378299; doi:10.1002/elps.70098)
Supplement: Supplementary file 1 — Supporting File 1: elps70098‐sup‐0001‐SupMat‐Data‐1.docx. [file ELPS-47--s002.docx]

**Development of Pentaplex Reverse Transcription Droplet Digital PCR Assay for Simultaneous Detection and Absolute Quantification of HIV-1, HIV-2, HCV, and HBV with Internal Control**

Soo Yeon Lim^1^, Un Na Koh^1^, Ah Leum Kim^1,^ Yebin Kim^1^, Ga Eun Kim^1^, Si-Keun Lim^1,2,3*^

^1^ Department of Forensic Sciences, Sungkyunkwan University, Suwon, Republic of Korea

^2^ ID-Cell Forensics Co., Sungkyunkwan University, Suwon, Republic of Korea

^3^ Convergence Bio Forensic Institute(CBFI), Biomedical Institute for Convergence at Sungkyunkwan University(BICS), Sungkyunkwan University, Suwon, Republic of Korea

***Corresponding author**

Si-Keun Lim: [sikeun-lim@skku.edu](mailto:sikeun-lim@skku.edu)

**Table S1** Nucleotide sequence of the internal control (IC) RNA, a 400 bp artificial, non-homologous sequence synthesized in-house

| **Internal control sequence** |
| --- |
| AGCTGCGAGGCACGATCGGATAGCACGGATCGGACTAGGCTACGGATCGGCTTTCGGATCAGGTCTCGAGAATATTTCGCGAGCCTGATACAGGTATCTGTATTGCCTATGCGGACCATGCGTGTAGCGACTCGGAGGGAGCAGGTCGGATTCACGGCTCCGTCTCATGCTTATTCTTGCAATCTAGGAATTCGGCTGTCGATTCGAGCTACACTGGAGCTCGGCATTACGGGCATAGTACGACTAAGCGTGAGCTAGCGCTAAGCGGGTATAAATCGAGTCACTGATCGGCTAGAGCTTAGCGATTTCGGTATATACGGCACGAGGCTAAGGCTCATGAGGATATCGGGGCCTAGAGCAGGGATTATATCTCGATCGGAGCTATAGGCGCTAGCGATCG |

**Table S2** Agents and reaction volumes used for reproducibility testing in the optimized Digital LightCycler® dPCR System

| **Reagent** | **Volume (μL)** |
| --- | --- |
| Digital LightCycler® 5x DNA Master | 7 |
| Ultrapure water | 𝓍^a^ |
| Primer (forward/reverse (except IC primer)) | 0.78^b^ each |
| Probe (except IC probe) | 0.875^c^ |
| IC Primer (F,R) | 0.612^d^ each |
| IC Probe | 1.05^e^ |
| DNA | 𝓍 |
| **Total** | **35** |

^a^ x indicates variable volumes adjusted to achieve a final reaction volume of 35 μL

^b^ Final concentration, 900 nM

^c^ Final concentration, 250 nM

^d^ Final concentration, 700 nM

^e^ Final concentration, 300 nM

**Table S3** Comparison of quantitative results between the pentaplex RT-ddPCR assay and the external institution after unit conversion to copies/mL

| **NO.** | **Pentaplex Results** | | **External Results** | |
| --- | --- | --- | --- | --- |
|  | **Copies/reaction** | **Estimated copies/mL** | **IU/mL** | **Estimated copies/mL^*^** |
| **1** | 14.12 | 1210.286 | **4676** | 2556.588 |
| **2** | 18.08 | 1549.714 | 4141 | 2264.079 |
| **3** | 5.96 | 510.857 | 56440 | 30858.39 |
| **4** | 109.4 | 9377.143 | 11030 | 6030.618 |
| **5** | 21 | 1800 | 93010 | 50852.93 |
| **6** | 13.58 | 1164 | 52930 | 28939.31 |
| **7** | 24.2 | 2074.286 | 482.6 | 263.86 |
| **8** | 45.6 | 3908.571 | 15410 | 8425.369 |
| **9** | 33.4 | 2862.857 | 63290 | 34603.61 |
| **10** | 242 | 20742.86 | 13470 | 7364.68 |
| **11** | 3.74 | 320.571 | 11550 | 6314.926 |
| **12** | 8.86 | 759.428 | 19030 | 10404.59 |

^*^ IU/mL values were converted to copies/mL using a factor of 1 IU = 1.829 copies

**Table S4** Differences in experimental conditions and sample input volumes between the external institution and the present study

| **Parameter** | **Pentaplex Assay** | **External Institution** |
| --- | --- | --- |
| Nucleic acid extraction kit | QIAamp® Viral RNA Mini Kit (QIAGEN, Germany) | ExiPrep™ Dx Viral DNA/RNA Kit (Bioneer, Korea) |
| Plasma input (μL) | 140 | 400 |
| Elution volume (μL) | 45 | 50 |
| Post-cDNA synthesis volume (μL) | 90 | Not specified |
| PCR platform | QX600™ Droplet Digital™ PCR System (Bio-Rad, USA) | Exicycler™96 (Bioneer, Korea) |
| PCR assay | Assay developed in this study | AccuPower® HIV-1 Quantitative RT-PCR Kit (Bioneer, Korea) |
| PCR input volume (μL) | 5 | 50 |
| Relative input difference |  | ***~50-fold higher***  (PCR input volume) |
